# Which Biomarkers Reveal Neonatal Sepsis?

**DOI:** 10.1371/journal.pone.0082700

**Published:** 2013-12-18

**Authors:** Kun Wang, Vineet Bhandari, Sofya Chepustanova, Greg Huber, Stephen O′Hara, Corey S. O′Hern, Mark D. Shattuck, Michael Kirby

**Affiliations:** 1 Department of Mathematics, Colorado State University, Fort Collins, Colorado, United States of America; 2 Department of Mechanical Engineering and Materials Science, Yale University, New Haven, Connecticut, United States of America; 3 Division of Perinatal Medicine, Department of Pediatrics, Yale University School of Medicine, New Haven, Connecticut, United States of America; 4 Kavli Institute for Theoretical Physics, University of California, Santa Barbara, California, United States of America; 5 Department of Mechanical Engineering & Materials Science, Department of Applied Physics, and Department of Physics, Yale University, New Haven, Connecticut, United States of America; 6 Benjamin Levich Institute and Physics Department, The City College of New York, New York, New York, United States of America; Technische Universität Dresden, Germany

## Abstract

We address the identification of optimal biomarkers for the rapid diagnosis of neonatal sepsis. We employ both canonical correlation analysis (CCA) and sparse support vector machine (SSVM) classifiers to select the best subset of biomarkers from a large hematological data set collected from infants with suspected sepsis from Yale-New Haven Hospital's Neonatal Intensive Care Unit (NICU). CCA is used to select sets of biomarkers of increasing size that are most highly correlated with infection. The effectiveness of these biomarkers is then validated by constructing a sparse support vector machine diagnostic classifier. We find that the following set of five biomarkers capture the essential diagnostic information (in order of importance): Bands, Platelets, neutrophil CD64, White Blood Cells, and Segs. Further, the diagnostic performance of the optimal set of biomarkers is significantly higher than that of isolated individual biomarkers. These results suggest an enhanced sepsis scoring system for neonatal sepsis that includes these five biomarkers. We demonstrate the robustness of our analysis by comparing CCA with the Forward Selection method and SSVM with LASSO Logistic Regression.

## Introduction

The identification and treatment of sepsis continues to be a major health issue. The incidence of sepsis is particularly high in the neonatal population, where low birth weight and other compromising factors make it a primary cause of morbidity and death [1]–[3]. Early identification and treatment are critically important to healthy patient outcomes given the inconsistent presentation of sepsis in terms of body temperature, which may be either above or below normal [4]–[6].

The most reliable diagnostic of neonatal sepsis, often referred to as the *gold standard*, is a blood culture test for bacteria. While this test is the most reliable available, it can take 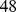 hours to obtain the results. As a result, treatment must often begin before the results are known. An additional complication is the fact that the blood culture test can be negative for one in five subjects with sepsis [2], [7]. Thus, it is of critical importance to identify new biomarkers that will enable fast and reliable hematological scoring systems for sepsis in its earliest stages.

The current hematological scoring system was first proposed by Rodwell, et al. in 1988 and is based on the following seven quantities: total leukocyte (or White Blood Cell, WBC) count, mature neutrophil count (also named Segs, Absolute Neutrophil Count, or ANC), immature neutrophil count (also named Bands, Absolute Band Count, or ABC), Immature to Total neutrophil count ratio (IT-ratio), Platelet count (Plt), and adverse changes in the total neutrophil count [8]. Another scoring system was proposed in Ref. [6] that characterizes a patient as septic if any two of the following four criteria are satisfied:

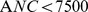
 or 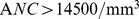

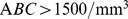

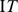
-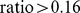

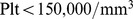
.

These hematological scores are supplemented by other observational evidence and measurements collected by physicians including body temperature, blood pressure, and clinical presentation in determining the course of treatment before the blood culture results are available.

Additional diagnostic hematological biomarkers have been studied such as C-reactive protein (CRP) [9], [10] and procalcitonin [11], [12]. While these biomarkers have shown to be correlated with sepsis, they are considered to have limited diagnostic information [4], [13]. More recently, the blood biomarker neutrophil CD64 has proved to be particularly promising for early detection of sepsis [14]–[16]. Neutrophil surface CD64 expression is a high affinity Fc receptor for immunoglobulin G (IgG) expressed on neutrophils (and other white blood cells). Quantities of CD64 increase markedly when neutrophils are activated by the human body's response to infection, and in particular, to sepsis.

The challenge of biomarker identification is reflected by the fact that over 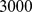 sepsis biomarker studies have been published with almost 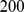 candidate biomarkers evaluated [4]. Nonetheless, clinicians are unsatisfied with the diagnostic tools currently available for making accurate and timely sepsis diagnoses that would also support appropriate therapies. The challenge is not to identify single biomarkers that pass a univariate test for diagnostic efficacy, but to determine which sets of biomarkers, when considered as a group, yield the most accurate prognosticator.

In this investigation, we integrate two tools for discovering information in large data sets. Embedded feature selection using a sparse support vector machine classifier [17], [18] and canonical correlation analysis [19], a tool for identifying relationships between two sets of variables. This two-pronged analysis provides a powerful general tool for the identification of biomarkers useful for multivariate scoring systems.

In this manuscript, we present a systematic study of the multivariate diagnostic capacity of a set of ten hematological biomarkers. Our goal is to establish a general approach that can be used effectively on potentially much larger sets of biomarkers. We develop an approach to identify a minimum set of predictive biomarkers with the ultimate goal of improving the early detection of sepsis. We verify the results by conducting an exhaustive evaluation of all possible combinations of biomarkers. We envision that the algorithms proposed here will be helpful tools as advances in biomedicine produce additional candidate biomarkers arising from new proteomic and metabolomic tests [20], [21].

## Results

A total of 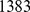 sepsis evaluations were performed on 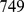 neonates during the study period. Blood cultures, complete blood counts (CBC), and neutrophil CD64 data were obtained for 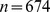 of the sepsis evaluations. One evaluation was excluded due to the high neutrophil CD64 value that skewed the results. Evaluations were partitioned into three groups: (1) blood culture positive septic group (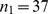), (2) clinically probable septic group (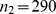), and (3) nonseptic group (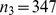). In this study, we combined groups 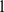 and 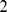 and labeled these subjects as having sepsis. Our analysis is based on the comparison between this combined septic group (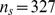) and nonseptic group (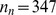). See Materials and Methods for details.

Data for ten hematological biomarkers were analyzed in this study including: (1) Age, (2) WBC count, (3) Hemoglobin count (Hgb), (4) Hematocrit percentage (Hct), (5) Plt, (6) Segs, (7) Bands, (8) Lymphocyte (Lymph) count as a percentage of WBC, (9) Monocyte (Mono) count as a percentage of WBC, and (10) neutrophil CD64 expression. Following Ref. [5], P-values were computed for the biomarker data and all ten biomarkers were determined to have predictive capacity.

### Optimal Subsets of Biomarkers

Multivariate correlation analysis is a general tool for exploring how variables are inter-related. Canonical correlation analysis provides a powerful tool for discovering relationships between two sets of variables. Given two sets of variables, CCA can identify subsets of each set, which when combined as latent variables, produce the maximum correlation between the two sets. In this study, we choose one set of variables to be the sepsis score, and the second set is taken from all possible subsets of the ten biomarkers. CCA can thus generate an ordered list of biomarkers that are most correlated with the sepsis score. See Materials and Methods for details.

Here we discuss the results of applying CCA to select the best combinations of sepsis biomarkers. We first consider the single biomarker with highest correlation to the sepsis score. As shown in Table 1, this biomarker is Bands. If we consider all pairs of biomarkers, Bands and Plt possess the highest correlation with sepsis score. We note that CD64 has the second highest correlation with sepsis score, in the univariate sense, but improves the correlation of Bands to sepsis score less than Plt, which has a lower univariate correlation with sepsis score. This is due to the fact that Bands and CD64 are more correlated than Bands and Plt, and so less information is provided by adding CD64. Hgb enters at 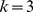 even though it has a very weak pairwise correlation with the sepsis score given it also has very weak pairwise correlation with Bands and Plt. The correlation saturates at 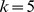 with the following combination set of biomarkers: Bands, CD64, Segs, WBC, and Plt. The rest of the biomarkers do not provide significant additional information about the sepsis score. The above analysis suggests that these five variables should be included in our sepsis scoring system.

**Table 1:** Comparison of the Canonical Correlation Analysis and Forward Selection of the biomarkers.

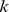 -combination	Correlation	Enter	Leave	Forward Selection
1	0.563	Bands		Bands
2	0.615	Plt		Plt
3	0.633	Hgb		Hgb
4	0.643	CD64		CD64
5	0.653	Segs, WBC	Hgb	Segs
6	0.660	Hgb		WBC
7	0.663	Age		Age
8	0.664	Lymph		Hct
9	0.666	Mono		Lymph
10	0.668	Hct		Mono

By applying CCA for all possible 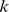-combinations 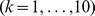, the subset of 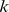 biomarkers with the highest correlation with the sepsis score is determined. The ‘Enter’ column indicates which biomarker is added to achieve the highest correlation at each 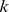. The ‘Leave’ column indicates which biomarker is eliminated from the combination at that particular 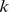. A biomarker will stay in the combination until it occurs in ‘Leave’ column. For instance, for the 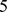-combination, the most correlated biomarkers include Bands, Plt, CD64, Segs, and WBC. Hgb, which was present in the 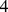-combination, is replaced by Segs and WBC at level 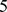. The ‘Forward Selection’ column is the biomarker selected by the forward selection method when applied one biomarker at a time.

#### Comparison with Forward Selection Method

Forward Selection (FS) is a well known data-driven selection method, where additional variables are added in one-by-one to improve the model [22]. The FS method selects the single variable out of the remaining set that gives the highest absolute correlation with the residual vector [23]. The results from FS on the sepsis data set are compared with those from CCA in Table 1. Both methods involve linear correlations, but FS is a greedy algorithm, which only produces a locally optimal solution. However, we find that up to 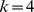, CCA and FS select the same subset of biomarkers. At 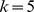, CCA and FS differ. Since FS can only select one feature at a time, at 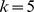, FS selects Segs, while CCA selects Segs and WBC and replaces Hgb. The manner in which we implemented CCA ensures a globally optimal solution for each 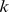.

In the next section, we validate this result using a classifier to predict the sepsis score in terms of these biomarkers.

### The Diagnostic Classifier

We seek to construct a decision function from the biomarker data that serves as a hematological scoring system, *i.e.* a function that maps a sample vector of biomarkers to a positive or negative sepsis diagnosis. Using the biomarkers identified by CCA above, WBC, Plt, Segs, Bands, and CD64, we propose the linear decision function: 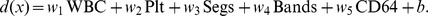



From the sparse support vector machine approach described in Materials and Methods, we determined the optimal decision function to be 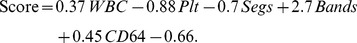
(1)


See Table 2 for the weights, 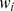, and their errors, and means and standard deviations of the biomarkers. With this decision function, if the Score is greater than or equal to zero the diagnosis is positive for sepsis, whereas if the Score is less than zero, the diagnosis is healthy or aseptic disease. We note that since the range of values of the biomarkers varies widely, all values of the biomarkers are normalized by subtracting the mean over all cases and then dividing by the standard deviation.

**Table 2:** Parameters for the classifier at k = 5.

m	Biomarker	Mean 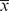	Standard Deviation 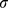	Weight 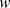	Standard Error of Weight 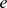
1	WBC	14.04	8.70	0.373	0.009
2	Plt	231.37	103.38	−0.876	0.012
3	Segs	39.64	17.25	−0.699	0.008
4	Bands	7.92	9.61	2.691	0.018
5	CD64	2.96	2.42	0.446	0.012

The parameters of the classifier for the sepsis score given in Equation (1), including the standard errors 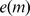 for each biomarker weight 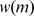.

The results of applying the classifier in Equation (1) to the full sepsis dataset are shown in Table 3. We calculated the true positive rate (TPR), true negative rate (TNR), positive predictive value (PPV), negative predictive value (NPV), and accuracy (ACC) (defined in Materials and Methods) for these five biomarkers. We emphasize that there are two remaining questions of interest. How good is the classifier? Did we identify the most predictive biomarkers from the original set of ten? We focus on the validation of these biomarkers in the next section.

**Table 3:** Performance of the classifier at k = 5 for SSVM and LLR.

Method	TPR	TNR	PPV	NPV	ACC
SSVM	0.838	0.905	0.893	0.856	0.875
LLR	0.740	0.960	0.945	0.797	0.853

Prediction measures for the classifier at k = 5 built by SSVM and LLR: true positive rate (TPR), true negative rate (TNR), positive predictive value (PPV), negative predictive value (NPV), and accuracy (ACC).

### Biomarker Validation

In this section, we have two goals. First, we will verify that the number of biomarkers suggested by CCA, 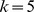, is optimal. Secondly, we seek to provide evidence that the CCA-selected biomarkers are optimal. To do this, we will perform an exhaustive analysis of all possible scoring systems for the ten biomarkers. Clearly this approach is not feasible for large sets of biomarkers, but we exploit the fact that we only have ten to illustrate the power of CCA biomarker selection by constructing all possible SSVM classifiers. We used the accuracy of the resulting decision functions for our validation.

#### Validation of the 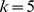 Classifier

**Figure 1:**
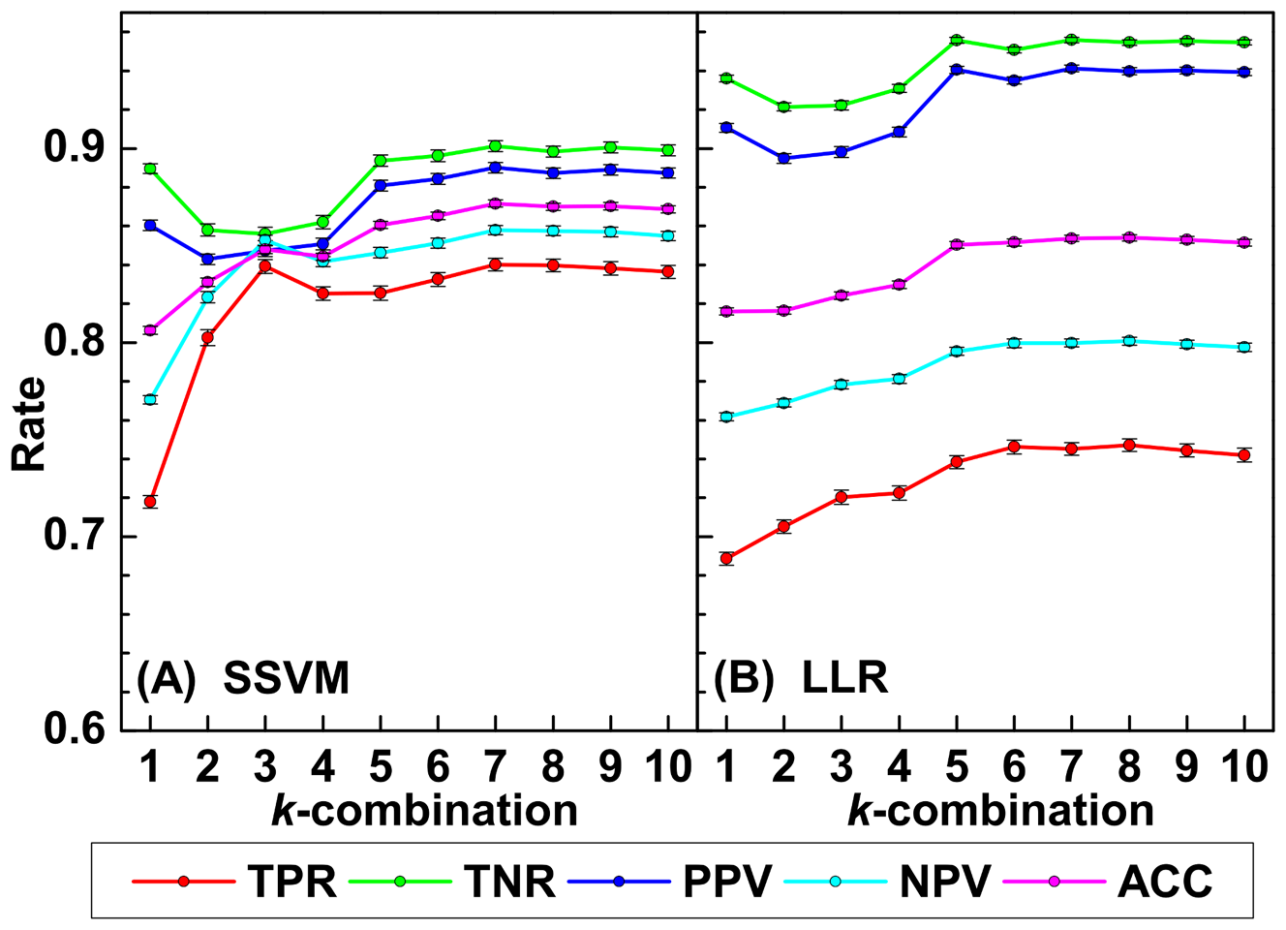
Prediction measures obtained from the (A) Sparse Support Vector Machine and (B) LASSO Logistic Regression methods. True positive rate (TPR), true negative rate (TNR), positive predictive value (PPV), negative predictive value (NPV), and accuracy (ACC) are shown for each 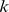-combination of biomarkers selected.

For each 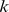, we select the 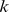-combination set of biomarkers as identified by CCA and shown in Table 1. We construct a decision function for each 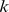 from 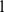 to 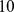 and evaluate several measures of the quality of the scoring system in Fig. 1. We find that each measure begins to saturate near 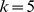, although one could argue that some slight improvement could be obtained by adding one or two more biomarkers for the given model. (We note that this particular model was not optimized over variations in the parameter 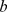.)

**Figure 2:**
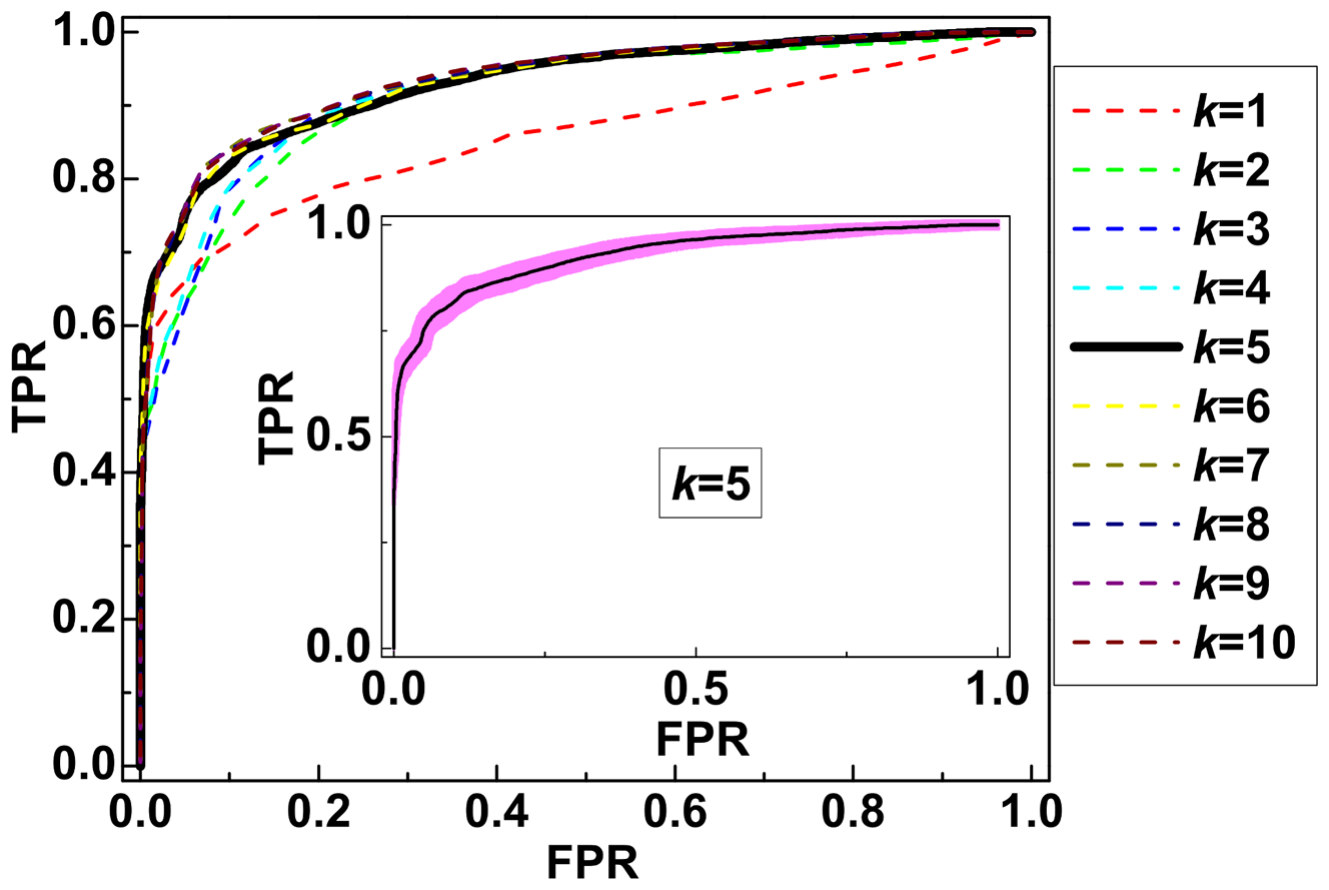
Receiver operating characteristic (ROC) curves. ROC curves of TPR versus FPR for optimal sets of 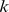 biomarkers where 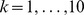 averaged over 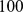 SSVM models. The shaded region in the inset shows the standard deviation for 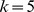.

Receiver operating characteristic (ROC) curves for true positive versus false positive rate provide additional insight into the determination of the minimal number of biomarkers that provide predictive information about sepsis infection. In Figure 2, we show that the ROC curves become independent of 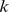 for 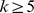, and thus 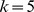 is indeed the appropriate number of biomarkers. In the inset to Figure 2, we show the ROC curve for 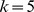 averaged over 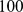 SSVM models.

#### Validation of the CCA Selected Biomarkers

**Figure 3:**
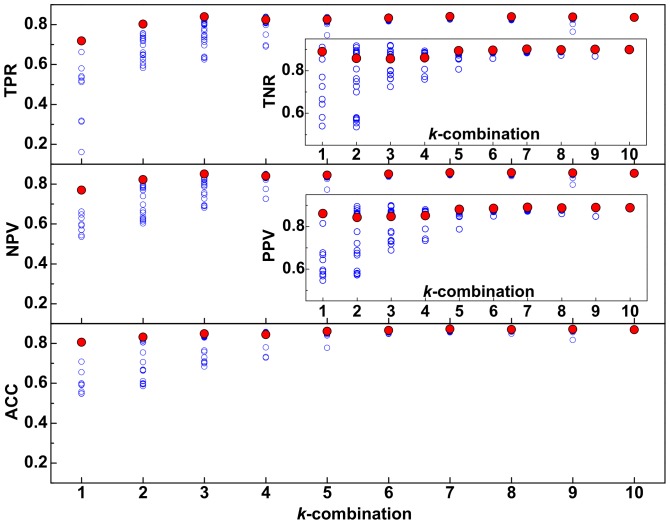
Exhaustive evaluation of statistical measures. The 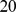 highest TPR, TNR, PPV, NPV, ACC values when SSVM was applied for all possible combinations of 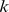 biomarkers (blue circles) from 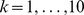. The solid red circles are the values for models built using the best 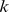 biomarkers selected by CCA.

We provide further evidence that our CCA biomarker selection was in fact the optimal one by applying SSVM to all possible combinations of biomarkers for each 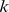. We show the TPR, TNR, PPV, NPV, and ACC for the top 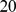 of all possible combinations in Figure 3. It is clear that the CCA-selected biomarkers possess the best statistical measures for each 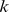.

#### Comparison with Logistic Regression

Logistic Regression (LR) is widely used for classification problems. A LR model can predict the outcome variable, such as the disease state ( *i.e.* sick or healthy) [24], by the new predictor inputs. The LASSO (Least Absolute Shrinkage and Selection Operator) algorithm [25] is a 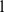-norm regularized logistic regression, which is extensively used for feature selection. By the 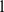-norm penalty, LASSO Logistic Regression (LLR) can achieve a sparse solution and exhibit a significantly high tolerance to the presence of many irrelevant features [26].

Here we also construct a LLR based classifier for each 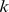 from 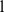 to 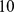 and plot the same statistical measures of the performance of the diagnostic system in Figure 1 as for SSVM. The sets of biomarkers determined by CCA are used for each 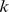-combination. We observe a similar saturation for all of the measures near 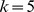. On our test data set we observe that LLR has a superior true negative rate while its true positive rate is inferior. The variability of the measures is substantially wider for LLR than SSVM (see Table 3 for the LLR and SSVM performance of the classifier at k = 5). In practice physicians may be concerned with a specific measure, e.g., negative predictive value. In this case, either of these methods could be used to optimize the negative predictive value. Please see Supporting Information Text S1 for more details.

## Materials and Methods

The data sets were obtained from a prospective study conducted in the Neonatal Intensive Care Unit at Yale-New Haven Hospital [27].This study was approved by the Yale University School of Medicine Human Investigation Committee. Consecutive patients, who underwent a sepsis work-up as deemed necessary by the attending neonatologist during the time period 1/2008-6/2009, were enrolled in the study [27].

### Sepsis Evaluations

The clinical and historical features used to identify patients at risk for sepsis include one or more of the following, as determined by the attending neonatologist [6], [28], [29]: (1) respiratory compromise (*e.g.* tachypnea, increase in frequency or severity of apnea, or increased ventilator support); (2) cardiovascular compromise (*e.g.* increased frequency or severity of bradycardic episodes, pallor, decreased perfusion, or hypotension); (3) metabolic changes (*e.g.* temperature instability, feeding intolerance, glucose instability, or metabolic acidosis); (4) neurological changes (*e.g.* lethargy, hypotonia, or irritability); and (5) antenatal risk factors (*e.g.* maternal Group B Streptococcus (GBS) colonization without adequate intrapartum prophylaxis, unknown maternal GBS status, maternal temperature, chorioamnionitis, preterm labor, or prolonged rupture of membranes). After the sepsis evaluation was performed, we utilized the following values derived from CBC to assign a sepsis score [8], [14]: (1) Absolute Neutrophil Count (ANC) 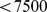 or 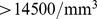; (2) Absolute Band Count (ABC) 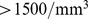; (3) Immature to Total neutrophil ratio (IT-ratio) 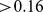; and (4) Platelet (Plt) count 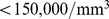. Infants who met 2 or more of these laboratory criteria were categorized as having a positive sepsis score. Hemoglobin was measured in the clinical hematology laboratory using a calorimetric method. The hematocrit was calculated after measuring the total red blood cell count (RBC) and the mean corpuscular volume (MCV) of the RBCs. All blood cultures were collected using standard sterile techniques. As per unit protocol, we attempt to obtain 2 blood cultures with a minimum of 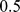 ml. The BACTEC (Becton Dickinson and Co., Sparks, MD) microbial detection system was used to detect positive blood cultures.

Neutrophil CD64 expression was measured using 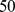 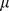
l of whole blood incubated for 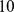 minutes at room temperature with a saturating amount of fluorescein isothiocyanate (FITC)-conjugated anti-CD64 monoclonal antibody or isotype control (Leuko64 kit, Trillium Diagnostics, Scarborough, ME), followed by ammonium chloride-based red cell lysis. Samples were washed once and re-suspended in 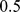 ml of phosphate-buffered saline with 0.1% bovine serum albumin. Flow cytometric analysis was accomplished using a Becton-Dickinson FACScan (Mountainview, CA) to collect log FITC fluorescence, log right-angle side scatter and forward scatter on a minimum of 50,000 leukocytes. Interassay standardization and neutrophil CD64 quantification were performed using FITC calibration beads (Leuko64 kit). Data analysis was performed using light scatter gating to define the neutrophil population, and the neutrophil CD64 Index was quantified as mean equivalent soluble fluorescence units using QuickCal for Winlist (Verity Software House, Topsham, ME) with a correction for nonspecific antibody binding by subtracting values for the isotype control [14]. This was expressed as an absolute value. Investigators checking and confirming the neutrophil CD64 results were blinded to the clinical data, including the blood culture results. Clinicians did not have access to the neutrophil CD64 values and these were not used to decide initiation or duration of antibiotic therapy.

### Evaluation Studied

Evaluations were obtained by accessing the electronic medical record from January 2008 through June 2009. Each evaluation typically included a CBC, two peripheral blood cultures, and other optional cultures. A patient could undergo multiple sepsis evaluations during admission. Since a single evaluation represented a separate episode of suspected sepsis and could be treated independently, we therefore treated all evaluations equivalently in this manuscript. Evaluations were excluded if the CBC, neutrophil CD64, or blood culture tests were not provided in the patient record. A total of 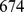 sepsis evaluations with complete hematologic, neutrophil CD64, and blood culture data were used for the analyses. (One evaluation, which was positive for 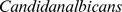 was excluded due to the high neutrophil CD64 value that skewed the results.) Information about each sepsis evaluation included (1) sepsis diagnosis type, (2) day of life that the evaluation was performed, and (3) CBC data and neutrophil CD64 expression. Ten biomarkers were included in the analysis: Age, WBC, Hgb, Hct, Plt, Segs, Bands, Lymph, Mono, and CD64. Additional details about the laboratory and clinical data were recently published [27], [30].

### Defining Sepsis Outcome

Individual sepsis evaluations with positive blood cultures were diagnosed as culture-proven sepsis according to the current National Healthcare Safety Network definitions for laboratory-confirmed bloodstream infections [31]. Individual sepsis evaluations with positive sepsis scores were categorized as clinical sepsis [8], [14]. This might include infants with other infectious diagnoses that were not accompanied by a positive blood culture, such as pneumonia, urinary tract infection, and necrotizing enterocolitis.

Three groups of evaluations were defined, and each evaluation was assigned to only one of the three groups. Group 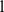 consisted of 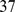 evaluations with a positive blood culture. Group 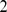 with “suspected sepsis” consisted of 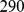 evaluations, where the patients lacked a definitive positive blood culture, but the clinical diagnosis was unable to rule out bacterial infection. Group 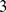 consisted of 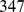 evaluations, for which either the blood culture or clinical diagnosis showed no evidence of infection.

### Data Preprocessing

First, each evaluation 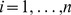, with data 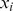, is categorized as septic (groups 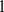 and 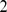 above) and nonseptic (group 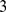), where 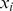 is a real-valued vector with 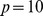 components (biomarkers) and 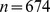 is the total number of evaluations. For convenience, we labeled each evaluation using the variable 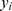, where 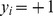 is the label for the septic group and 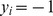 is given to each in the nonseptic group. To standardize the range of independent biomarkers, we normalized the real-valued data 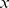, a 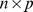 matrix, to have zero mean and unit standard deviation for each biomarker [32]: 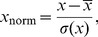
(2)where 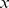 and 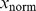 are 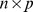 matrices, 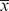 and 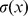 are the mean value and standard deviation of 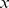 for each biomarker.

### Canonical Correlation Analysis

CCA is a multivariate statistical tool that facilitates the study of interrelationships among multiple variables [19], [33]. A linear combination of variables can be chosen by CCA such that the correlation between two sets of data is maximized [34]. In our studies, the two sets of data are the sepsis score 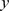 and each distinct 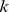-combination of the 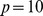 biomarkers in the data matrix 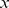. In this investigation, CCA is used to identify the set of 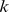-biomarkers most correlated as a group to the sepsis score. Please see Supplementary Text S1 for more details.

To explore the redundancy among the biomarkers, we calculated the correlations between each possible 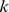-combination of 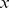 and 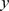 using CCA. By varying 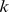 from 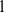 (single biomarker) to 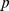 (all biomarkers), we selected the specific set of biomarkers that possessed the highest correlation with 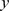 for each 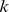. The sets of biomarkers that had the largest correlation with the sepsis score and their corresponding correlation coefficients are shown in Table 1.

### Sparse Support Vector Machines (SSVM)

We applied the SSVM ensemble method to build a classifier for each of the CCA-selected 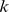-combination of biomarkers selected by CCA. A linear support vector machine (SVM) is a widely used classifier, which finds the hyperplane that separates high-dimensional data with maximum margin by categories. The search of this hyperplane can be translated into the following optimization problem: 
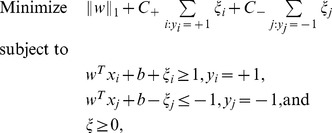
(3)where 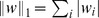 is the 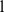-norm of a vector, which induces the sparsity in the weight vector 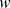 [17]. We refer to the solution of Equation (3) as a sparse support vector machine (SSVM) following Ref. [18]. Note that splitting the classes in the objective function allows for unbalanced sample sizes.

Due to the limited size and noise of our data, a bootstrap aggregation method was applied to build an ensemble of SSVM classifiers using the following procedure [35], [36]:

1. The data set 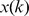 is randomly divided into a learning set 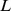 and a test set 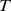. 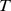 is one third of the data.
2. Based on the bootstrap aggregation method, a bootstrap training set 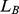 is randomly selected from the original learning set 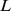 with replacement. That is, 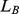 has the same number of samples as the original training set 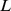, but with several training samples appearing multiple times. Each bootstrap set 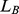 contains 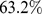 unique samples of the original training set 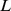. By repeating this process 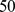 times, an ensemble of classifiers 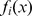, with 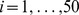, is built by the SSVM. To have the same total cost for both false positives and false negatives, the parameters 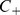 and 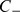 of the SSVM are chosen according to 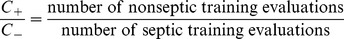 with 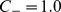 since the results are not sensitive to the overall scale of 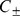.
3. The final classification is obtained by calculating the mean of the ensemble of 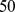 classifiers.
4. The random division of the data into 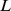 and 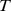 is repeated 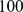 times, after which we calculate the mean and standard deviation. We used the same 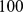 random divisions of the training and test sets for each 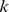.

### Calculation of Statistical Measures

The statistical measures of the performance of a classifier are measured using ACC, TPR, TNR, NPV, and PPV. For the sake of completeness, we include their definitions:
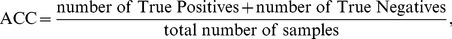
(4a)

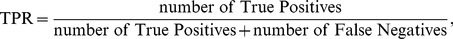
(4b)

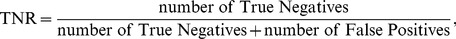
(4c)

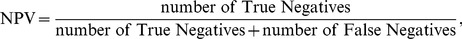
(4d)

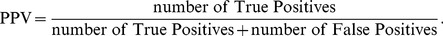
(4e)


Here, these statistical measures are calculated for each one of the 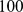 random divisions of test sets 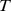 by the classifier built on the bootstrap aggregation method. Their mean and standard deviation are calculated from the groups obtained from the 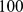 random divisions.

## Discussion

### Clinical Issues in Sepsis Diagnosis

The problems associated with confirming sepsis with positive blood cultures, as mentioned earlier, has led clinicians to investigate alternate approaches for confirmation of diagnosis of blood-cluster negative or clinical sepsis and prevention of missed or under treatment of neonates with antibiotics. Clinical parameters are notorious for their non-specific nature in detecting infection, especially in premature neonates; however, a scoring system based on a 7-item weighted clinical score has been suggested [37]. In real world settings, most clinicians rely on clinical judgment, in concert with specific hematological criteria, to identify infants with sepsis. The hematological criteria have usually included ANC, ABC, IT-ratio, and platelet counts, as was done in the present study [6], [14], [38]–[43]. Unfortunately, this approach has not proven very reliable due to the inherent subjective nature of the clinical assessment and the variability of the hematological parameters secondary to physiological derangements and non-infectious medical conditions, and has led to over-treatment of neonates.

It has been suggested that the addition of specific molecular markers might improve diagnostic accuracy of neonatal sepsis. Among the acute-phase reactants, CRP is probably the most well studied, but its value for diagnostic accuracy has had mixed results [7], [44]. Among the newer ones, procalcitonin [45]–[49] and neutrophil CD64 [14], [15] have shown promise. Neutrophil CD64 values have been reported to be sustained for at least 24 hours in neonates with sepsis [50].

Several studies have investigated the usefulness of the CD64 Index in the NICU population, albeit in much smaller cohorts, but with promising results in both the preterm and term populations, as well as in cases of both early-onset sepsis and late-onset sepsis [14], [51]–[56]. Recently, studies have suggested that the diagnostic accuracy of neutrophil CD64 is superior to the IT-ratio [57] and CRP [58] for the early detection of neonatal sepsis. Secondly, testing can be done on the same sample sent for a CBC evaluation as it requires only 50 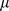
l of blood. Thirdly, the CD64 results can be made available within hours of the CBC, since most clinical laboratories in the developed and some developing countries have flow cytometry technology. Furthermore, standard cell counters which use flow cytometry have the potential to incorporate anti-CD64 antibodies and software to provide an even more rapid enumeration of CD64 indices nearly simultaneous with CBC results. Hence, we believe that a scoring system that can incorporate the common CBC parameters with the neutrophil CD64, as was done with our analyses, would provide objective criteria for recognizing neonatal sepsis and guidance for initiation and/or early termination of antibiotic therapy. Additional independent validation of our results is needed before incorporation of our diagnostic sepsis score can be recommended for routine clinical use.

### Identification of the Optimal Subset of Biomarkers

We have proposed a new approach for biomarker identification based on the integrated use of CCA and SSVM on a labeled data set. We found that for the neonatal sepsis data our approach produced the optimal set of 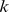 hematological biomarkers for all possible 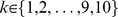. We validated our results by conducting an exhaustive search of all combinations of biomarkers and ranking them based on their classification accuracy. These results showed that our approach produced either the absolute top combination, or a combination of biomarkers with statistically indistinguishable performance. Although this study explored a relatively small set of biomarkers, the CCA approach can be applied to potentially much larger sets by exploiting the relative weighting of the features (see Equations S1 and S2) and selecting only the most important features. From Equation S3, CCA requires finding the pseudo-inverse of a 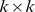 matrix, where 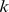 is the number of biomarkers. Even without invoking sparse methods, one can easily investigate systems with 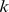 of order 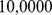.

Our approach identifies Bands as the most significant biomarker in our set for detecting neonatal sepsis. The next four most sigificant hematological biomarkers, in order of importance, appear to be Plt, neutrophil CD64, WBC, and Segs. We note that, as illustrated in Figure S1, the reason for the significance of Bands may be attributed to the fact that it is highly correlated with subjects with a negative diagnosis while much less correlated with subjects with a positive diagnosis. This could be related to the significant variation in Bands for sick individuals as evidenced by Table S1.

We explore LLR in addition to SSVM to corroborate our results. In each case we see that an exhaustive combinatorial evaluation of the classifiers determines that the CCA selected biomarkers were indeed optimal. See Figures S2 and S3 for a graphical summary of these numerical experiments. Additionally we found that the Forward Selection results were very similar to the biomarkers identified by CCA on this data set, i.e., Bands, Plt, Hgb, CD64 and Segs. CCA selected WBC and not Hgb for the best 5-combination. The classifiers using these two sets of biomarkers perform very similarly with a very slight edge to the CCA biomarkers. However, in general, forward selection is a greedy algorithm and it is possible that the sets of biomarkers identified by CCA and FS could be quite different. Classification algorithms such as SSVM or LLR can then assist in comparing and evaluating the selected biomarkers.

We propose that the results found in this investigation, in particular, the new sepsis scoring system, sets the stage for independent investigators to clinically validate these results using alternative sepsis databases. In particular, it will be interesting to ascertain whether this scoring system is also relevant for adults. It is also possible to envision modifying the scoring system based on new data related to alternative scenarios, e.g., septic adults infected by Gram negative microorganisms. Although we propose CCA in conjunction with SSVM as an approach for biomarker identification, the strength of the methodology lies in the exploitation of multivariate relationships within the data and other methods that do this also merit further exploration.

## Supporting Information

Figure S1
**Heatmaps of pairwise correlations magnitude.** The pairwise correlations were calculated for any pair of all 10 biomarkers in septic group (A) and nonseptic group (B). The biomarkers in both 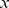-axis and 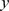-axis for all heatmaps are sorted ascending by the corrlation magnitude with sepsis score. The intensity of the color indicates the correlation magnitude in the pair associated with the corresponding labels of 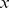-axis and 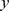-axis. A high magnitude implies a strong association between two variables.(TIF)

Figure S2
**Exhaustive evaluation of statistical measures.** The 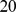 highest TPR, TNR, PPV, NPV, ACC values when LLR was applied for all possible combinations of 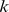 biomarkers (blue circles) from 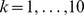. The solid red circles are the values for models built using the best 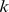 biomarkers selected by CCA.(TIF)

Figure S3
**Receiver operating characteristic (ROC) curves.** ROC curves of TPR versus FPR for optimal sets of 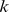 biomarkers where 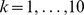 averaged over 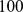 LLR models. The shaded region in the inset shows the standard deviation for 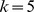.(TIF)

Table S1
**Characteristics of individual biomarkers by group.** Statistical analysis of individual biomarker based on the evaluation distributions of septic group and nonseptic group. Results are presented as mean (standard deviation). 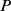 values are comparisons between septic group and nonseptic group. Any significance level of 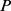 less than 0.05 was associated with the diagnosis.(PDF)

Text S1 **Supplementary Methods.**
(PDF)

## References

[1] Garcia-Prats JA, Cooper TR, Schneider VF, Stager CE, Hansen TN (2000) Rapid detection of microorganisms in blood cultures of newborn infants utilizing an automated blood culture system. Pediatrics 105: 523–527. 10699103 10.1542/peds.105.3.523

[2] Ganatra HA, Stoll BJ, Zaidi AK (2010) International perspective on early-onset neonatal sepsis. Clinics in Perinatology 37: 501–523. 20569819 10.1016/j.clp.2010.02.004

[3] Stoll BJ, Hansen N, Fanaroff AA, Wright LL, Carlo WA, et al (2002) Late-onset sepsis in very low birth weight neonates: the experience of the nichd neonatal research network. Pediatrics 110: 285–291. 12165580 10.1542/peds.110.2.285

[4] Charalampos P, Vincent JL (2010) Sepsis biomarkers: a review. Critical Care 14: R15. 20144219 10.1186/cc8872 PMC2875530

[5] Gibot S, Béné MC, Noel R, Massin F, Guy J, et al (2012) Combination biomarkers to diagnose sepsis in the critically ill patient. American journal of respiratory and critical care medicine 186: 65–71. 22538802 10.1164/rccm.201201-0037OC

[6] Gonzalez BE, Mercado CK, Johnson L, Brodsky NL, Bhandari V (2003) Early markers of late-onset sepsis in premature neonates: clinical, hematological and cytokine profile. Journal of perinatal medicine 31: 60–68. 12661146 10.1515/JPM.2003.009

[7] Benitz WE (2010) Adjunct laboratory tests in the diagnosis of early-onset neonatal sepsis. Clinics in Perinatology 37: 421–438. 20569816 10.1016/j.clp.2009.12.001

[8] Rodwell RL, Leslie AL, Tudehope DI (1988) Early diagnosis of neonatal sepsis using a hematologic scoring system. The Journal of pediatrics 112: 761–767. 3361389 10.1016/s0022-3476(88)80699-1

[9] Ng PC, Cheng SH, Chui KM, Fok TF, Wong MY, et al (1997) Diagnosis of late onset neonatal sepsis with cytokines, adhesion molecule, and c-reactive protein in preterm very low birthweight infants. Archives of Disease in Childhood-Fetal and Neonatal Edition 77: F221–F227. 9462194 10.1136/fn.77.3.f221 PMC1720722

[10] Da Silva O, Ohlsson A, Kenyon C (1995) Accuracy of leukocyte indices and creactive protein for diagnosis of neonatal sepsis: a critical review. The Pediatric infectious disease journal 14: 362–366. 7638010 10.1097/00006454-199505000-00005

[11] Monneret G, Labaune J, Isaac C, Bienvenu F, Putet G, et al (1997) Procalcitonin and c-reactive protein levels in neonatal infections. Acta Paediatrica 86: 209–212. 9055895 10.1111/j.1651-2227.1997.tb08870.x

[12] Hatherill M, Tibby SM, Sykes K, Turner C, Murdoch IA (1999) Diagnostic markers of infection: comparison of procalcitonin with c reactive protein and leucocyte count. Archives of disease in childhood 81: 417–421. 10519716 10.1136/adc.81.5.417 PMC1718133

[13] Malik A, Hui CP, Pennie RA, Kirpalani H (2003) Beyond the complete blood cell count and c-reactive protein: a systematic review of modern diagnostic tests for neonatal sepsis. Archives of pediatrics & adolescent medicine 157: 511. 12796229 10.1001/archpedi.157.6.511

[14] Bhandari V, Wang C, Rinder C, Rinder H (2008) Hematologic profile of sepsis in neonates: Neutrophil CD64 as a diagnostic marker. Pediatrics 121: 129. 18166566 10.1542/peds.2007-1308

[15] Ng PC, Li G, Chui KM, Chu WC, Li K, et al (2004) Neutrophil cd64 is a sensitive diagnostic marker for early-onset neonatal infection. Pediatric research 56: 796–803. 15371562 10.1203/01.PDR.0000142586.47798.5E

[16] Ng P, Li K, Wong P, Chui KM, Wong E, et al (2002) Neutrophil cd64 expression: a sensitive diagnostic marker for late-onset nosocomial infection in very low birthweight infants. Pediatric research 51: 296–303. 11861933 10.1203/00006450-200203000-00006

[17] Mangasarian OL (1999) Arbitrary-norm separating plane. Operations Research Letters 24: 15–23.

[18] Chepustanova S, Gittins C, KirbyM(2013) Band selection in hyperspectral imagery using sparse support vector machines. submitted.

[19] Mardia KV, Kent JT, Bibby JM (1980) Multivariate analysis.

[20] Srinivasan L, Harris MC (2012) New technologies for the rapid diagnosis of neonatal sepsis. Current Opinion in Pediatrics 24: 165. 22273634 10.1097/MOP.0b013e3283504df3

[21] Mussap M (2012) Laboratory medicine in neonatal sepsis and inflammation. Journal of Maternal-Fetal and Neonatal Medicine 25: 24–26. 10.3109/14767058.2012.715000 22958009

[22] Efroymson M (1960) Multiple regression analysis. Mathematical methods for digital computers 1: 191–203.

[23] Sjöstrand K, Clemmensen LH, Larsen R, Ersbøll B (2012) Spasm: A matlab toolbox for sparse statistical modeling. Journal of Statistical Software Accepted for publication.

[24] Milbrandt EB, Clermont G, Martinez J, Kersten A, Rahim MT, et al (2006) Predicting late anemia in critical illness. Crit Care 10: R39. 16507173 10.1186/cc4847 PMC1550792

[25] Tibshirani R (1996) Regression shrinkage and selection via the lasso. Journal of the Royal Statistical Society Series B (Methodological): 267–288.

[26] Ng AY (2004) Feature selection, l 1 vs. l 2 regularization, and rotational invariance. In: Proceedings of the twenty-first international conference on Machine learning. ACM, p. 78.

[27] Streimish I, Bizzarro M, Northrup V, Wang C, Renna S, et al (2012) Neutrophil cd64 as a diagnostic marker in neonatal sepsis. The Pediatric infectious disease journal 31: 777–781. 22481422 10.1097/INF.0b013e318256fb07 PMC3375383

[28] Klinger G, Levy I, Sirota L, Boyko V, Reichman B, et al (2009) Epidemiology and risk factors for early onset sepsis among very-low-birthweight infants. American journal of obstetrics and gynecology 201: 38–e1. 19380122 10.1016/j.ajog.2009.03.006

[29] Benitz WE, Gould JB, Druzin ML (1999) Risk factors for early-onset group b streptococcal sepsis: estimation of odds ratios by critical literature review. Pediatrics 103: e77. 10353974 10.1542/peds.103.6.e77

[30] Streimish I, Bizzarro M, Northrup V, Wang C, Renna S, et al.. (2013) Neutrophil cd64 with hematologic criteria for diagnosis of neonatal sepsis. American Journal of Perinatology. 10.1055/s-0033-1334453 23456906

[31] Horan TC, Andrus M, Dudeck MA (2008) CDC/NHSN surveillance definition of health care–associated infection and criteria for specific types of infections in the acute care setting. American journal of infection control 36: 309–332. 18538699 10.1016/j.ajic.2008.03.002

[32] Morik K, Brockhausen P, Joachims T (1999) Combining statistical learning with a knowledge-based approach-a case study in intensive care monitoring. In: Machine Learning-International Workshop Then Conference. Morgan Kaufmann Publishers, Inc., pp. 268–277.

[33] Tofallis C (1999) Model building with multiple dependent variables and constraints. Journal of the Royal Statistical Society: Series D (The Statistician) 48: 371–378.

[34] Björck A, Golub GH (1973) Numerical methods for computing angles between linear subspaces. Mathematics of computation 27: 579–594.

[35] Breiman L (1996) Bagging predictors. Machine learning 24: 123–140.

[36] Dietterich TG (2000) Ensemble methods in machine learning. In: Multiple classifier systems, Springer. pp. 1–15.

[37] Singh SA, Dutta S, Narang A (2003) Predictive clinical scores for diagnosis of late onset neonatal septicemia. Journal of tropical pediatrics 49: 235–239. 12929886 10.1093/tropej/49.4.235

[38] Manucha V, Rusia U, Sikka M, Faridi M, Madan N (2002) Utility of haematological parameters and c-reactive protein in the detection of neonatal sepsis. Journal of paediatrics and child health 38: 459–464. 12354261 10.1046/j.1440-1754.2002.00018.x

[39] Nigro KG, ORiordan M, Molloy EJ, Walsh MC, Sandhaus LM (2005) Performance of an automated immature granulocyte count as a predictor of neonatal sepsis. American journal of clinical pathology 123: 618–624. 15743752 10.1309/73H7-K7UB-W816-PBJJ

[40] Kudawla M, Dutta S, Narang A (2008) Validation of a clinical score for the diagnosis of late onset neonatal septicemia in babies weighing 1000–2500 g. Journal of tropical pediatrics 54: 66–69. 17698886 10.1093/tropej/fmm065

[41] Waliullah S, Islam M, Siddika M, Hossain M, Jahan I, et al (2010) Evaluation of simple hematological screen for early diagnosis of neonatal sepsis. Mymensingh medical journal: MMJ 19: 41. 20046170

[42] Selimovic A, Skokic F, Bazardzanovic M, Selimovic Z (2010) The predictive score for early-onset neonatal sepsis. Turk J Pediatr 52: 139–144. 20560248

[43] Narasimha A, Kumar MH (2011) Significance of hematological scoring system (hss) in early diagnosis of neonatal sepsis. Indian Journal of Hematology and Blood Transfusion 27: 14–17. 22379289 10.1007/s12288-010-0050-2 PMC3102509

[44] Mannan M, Shahidullah M, Noor M, Islam F, Alo D, et al (2010) Utility of creactive protein and hematological parameters in the detection of neonatal sepsis. Mymensingh medical journal: MMJ 19: 259–263. 20395923

[45] Sastre JBL, Solís DP, Serradilla VR, Colomer BF, Cotallo GDC, et al (2006) Procalcitonin is not sufficiently reliable to be the sole marker of neonatal sepsis of nosocomial origin. BMC pediatrics 6: 16. 16709255 10.1186/1471-2431-6-16 PMC1526729

[46] Cetinkaya M, Özkan H, Köksal N, S Çelebi MH, et al (2008) Comparison of serum amyloid a concentrations with those of c-reactive protein and procalcitonin in diagnosis and follow-up of neonatal sepsis in premature infants. Journal of Perinatology 29: 225–231. 19078972 10.1038/jp.2008.207

[47] Naher B, Mannan M, Noor K, Shahidullah M (2011) Role of serum procalcitonin and c-reactive protein in the diagnosis of neonatal sepsis. Bangladesh Medical Research Council Bulletin 37: 40–46. 21877603 10.3329/bmrcb.v37i2.8432

[48] Fendler WM, Piotrowski AJ (2008) Procalcitonin in the early diagnosis of nosocomial sepsis in preterm neonates. Journal of paediatrics and child health 44: 114–118. 17927729 10.1111/j.1440-1754.2007.01230.x

[49] Vouloumanou EK, Plessa E, Karageorgopoulos DE, Mantadakis E, Falagas ME (2011) Serum procalcitonin as a diagnostic marker for neonatal sepsis: a systematic review and meta-analysis. Intensive care medicine 37: 747–762. 21380522 10.1007/s00134-011-2174-8

[50] Layseca-Espinosa E, Pérez-González LF, Torres-Montes A, Baranda L, De La Fuente H, et al (2002) Expression of cd64 as a potential marker of neonatal sepsis. Pediatric allergy and immunology 13: 319–327. 12431190 10.1034/j.1399-3038.2002.01064.x

[51] Ng PC, Lam HS (2010) Biomarkers for late-onset neonatal sepsis: cytokines and beyond. Clinics in perinatology 37: 599–610. 20813273 10.1016/j.clp.2010.05.005 PMC7119343

[52] Morsy A, Elshall L, Zaher M, Abd EM, Nassr A (2008) Cd64 cell surface expression on neutrophils for diagnosis of neonatal sepsis. The Egyptian journal of immunology/Egyptian Association of Immunologists 15: 53. 20306688

[53] Groselj-Grenc M, Ihan A, Derganc M (2008) Neutrophil and monocyte cd64 and cd163 expression in critically ill neonates and children with sepsis: comparison of fluorescence intensities and calculated indexes. Mediators of inflammation 2008. 10.1155/2008/202646 PMC2442385 18604302

[54] Groselj-Grenc M, Ihan A, Pavcnik-Arnol M, Kopitar AN, Gmeiner-Stopar T, et al (2009) Neutrophil and monocyte cd64 indexes, lipopolysaccharide-binding protein, procalcitonin and c-reactive protein in sepsis of critically ill neonates and children. Intensive care medicine 35: 1950–1958. 19756501 10.1007/s00134-009-1637-7

[55] Zeitoun AA, Gad SS, Attia FM, Abu Maziad AS, Bell EF (2010) Evaluation of neutrophilic cd64, interleukin 10 and procalcitonin as diagnostic markers of earlyand late-onset neonatal sepsis. Scandinavian journal of infectious diseases 42: 299–305. 20085423 10.3109/00365540903449832

[56] Dilli D, Oguz ŞS, Dilmen U, Köker MY, Kizilgün M (2010) Predictive values of neutrophil cd64 expression compared with interleukin-6 and c-reactive protein in early diagnosis of neonatal sepsis. Journal of clinical laboratory analysis 24: 363–370. 21089165 10.1002/jcla.20370 PMC6647582

[57] Davis BH, Bigelow NC (2005) Comparison of neutrophil cd64 expression, manual myeloid immaturity counts, and automated hematology analyzer flags as indicators of infection or sepsis. Laboratory Hematology 11: 137–147. 16024338

[58] Choo YK, Cho HS, Seo IB, Lee HS (2012) Comparison of the accuracy of neutrophil cd64 and c-reactive protein as a single test for the early detection of neonatal sepsis. Korean journal of pediatrics 55: 11–17. 22359525 10.3345/kjp.2012.55.1.11 PMC3282213

