# Supplementary figures and images for: Which Biomarkers Reveal Neonatal Sepsis?
Source: PLoS One. 2013 Dec 18;8(12):e82700. doi: 10.1371/journal.pone.0082700 (PMC3867385; doi:10.1371/journal.pone.0082700)

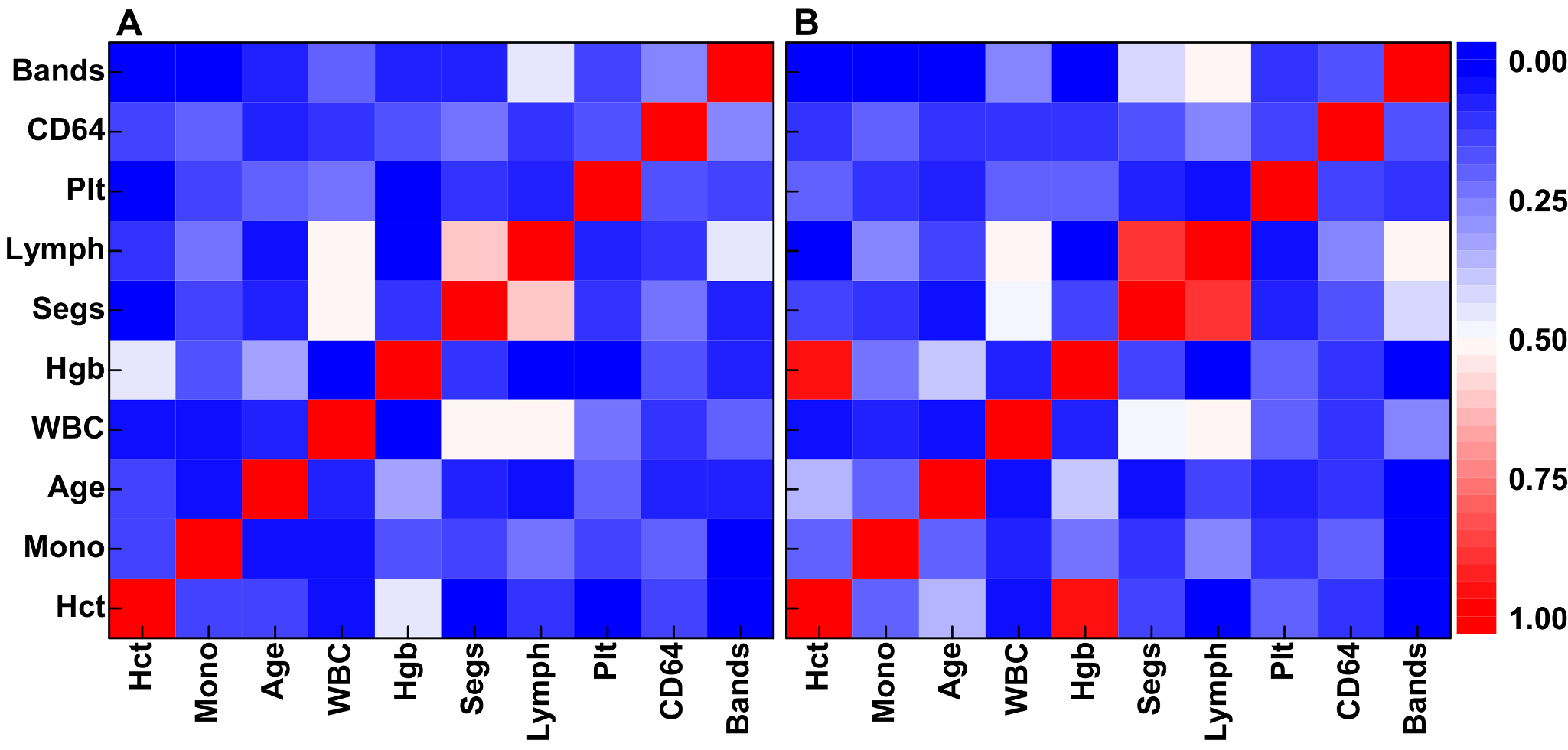

Supplement: Figure S1 — Heatmaps of pairwise correlations magnitude. The pairwise correlations were calculated for any pair of all 10 biomarkers in septic group (A) and nonseptic group (B). The biomarkers in both -axis and -axis for all heatmaps are sorted ascending by the corrlation magnitude with sepsis score. The intensity of the color indicates the correlation magnitude in the pair associated with the corresponding labels of -axis and -axis. A high magnitude implies a strong association between two variables. (TIF) [file pone.0082700.s001.tif]

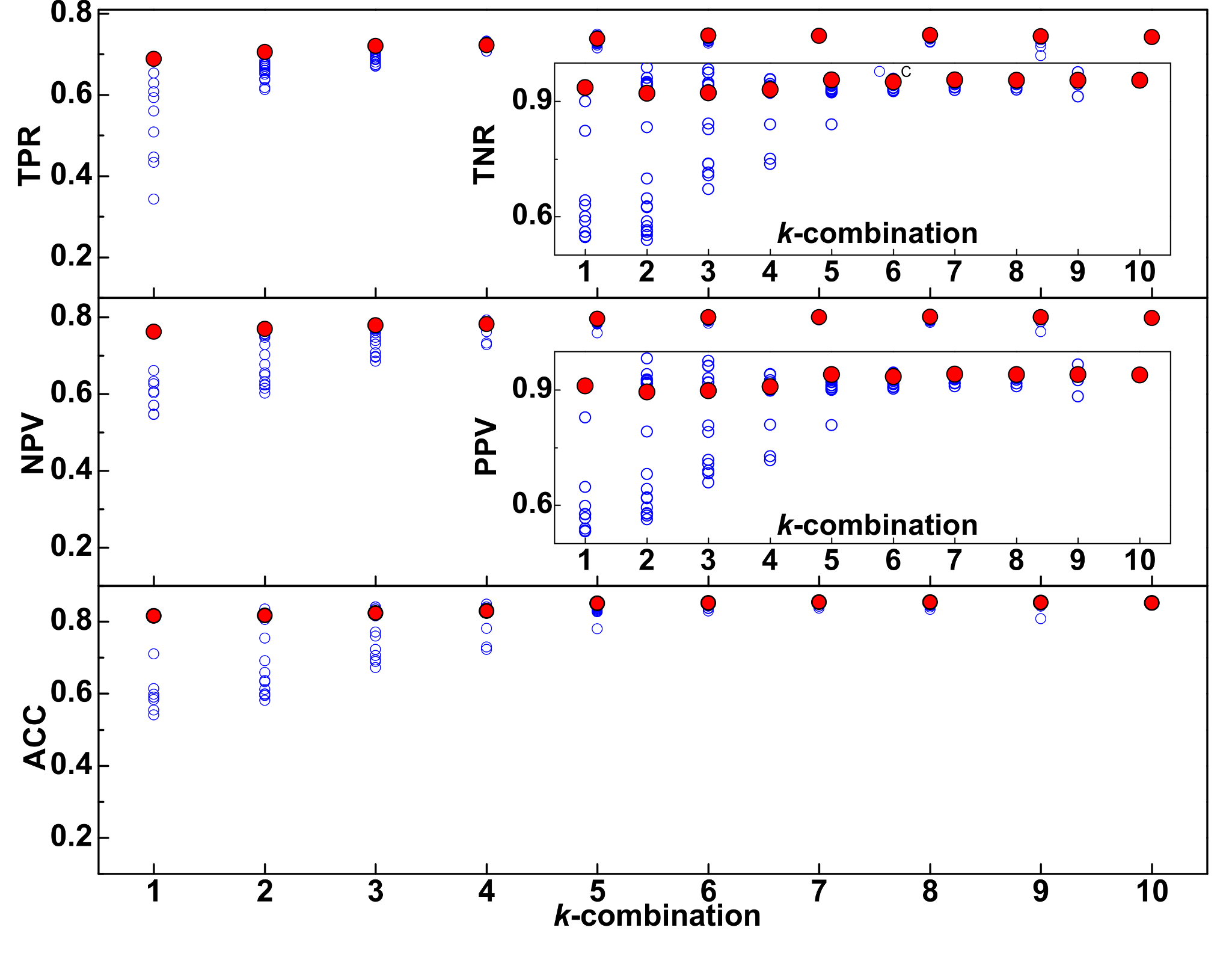

Supplement: Figure S2 — Exhaustive evaluation of statistical measures. The highest TPR, TNR, PPV, NPV, ACC values when LLR was applied for all possible combinations of biomarkers (blue circles) from . The solid red circles are the values for models built using the best biomarkers selected by CCA. (TIF) [file pone.0082700.s002.tif]

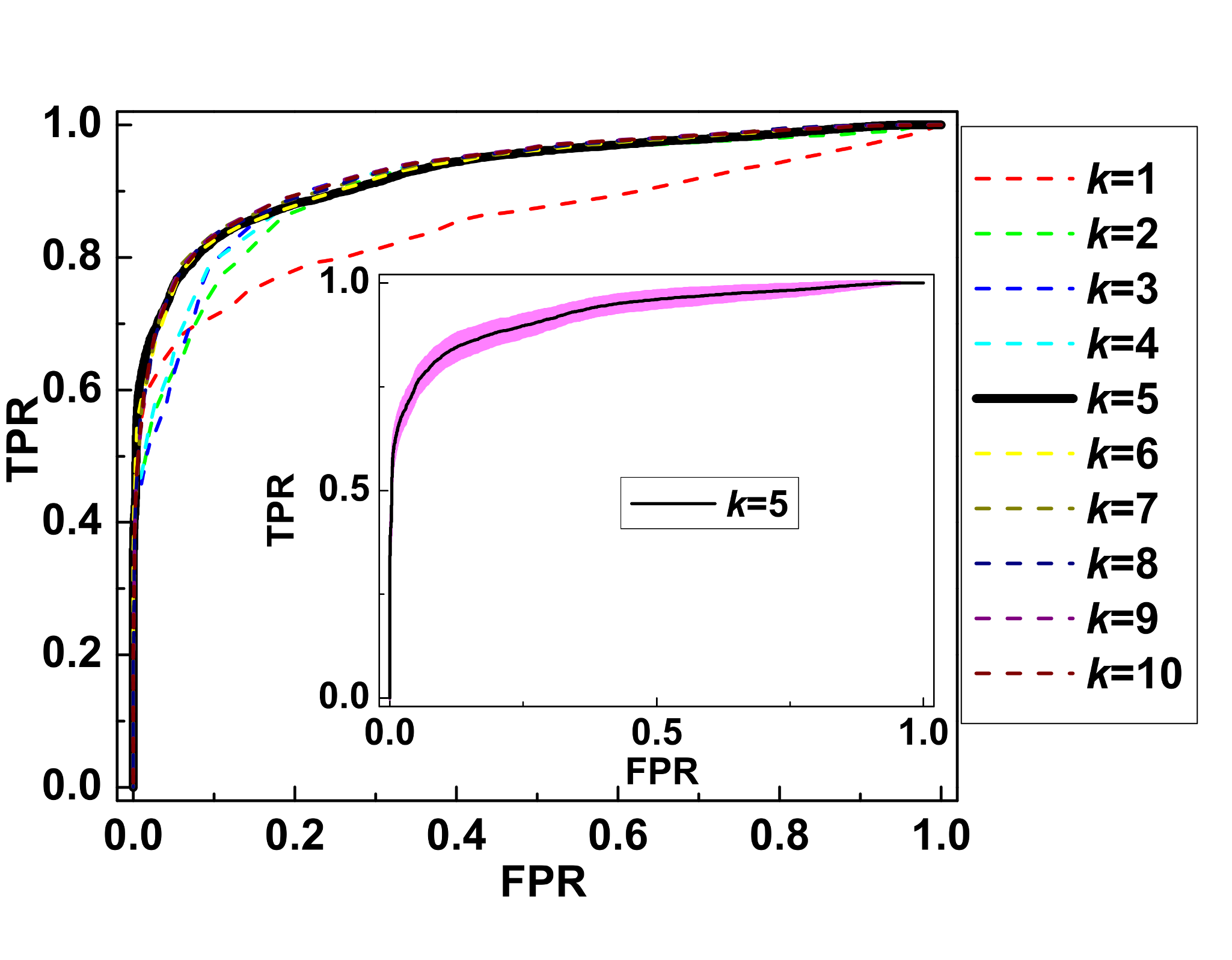

Supplement: Figure S3 — Receiver operating characteristic (ROC) curves. ROC curves of TPR versus FPR for optimal sets of biomarkers where averaged over LLR models. The shaded region in the inset shows the standard deviation for . (TIF) [file pone.0082700.s003.tif]
