# Supplementary material for: Which Biomarkers Reveal Neonatal Sepsis?
Source: PLoS One. 2013 Dec 18;8(12):e82700. doi: 10.1371/journal.pone.0082700 (PMC3867385; doi:10.1371/journal.pone.0082700)
Supplement: Table S1 — Characteristics of individual biomarkers by group. Statistical analysis of individual biomarker based on the evaluation distributions of septic group and nonseptic group. Results are presented as mean (standard deviation). values are comparisons between septic group and nonseptic group. Any significance level of less than 0.05 was associated with the diagnosis. (PDF) [file pone.0082700.s004.pdf]

Table S1. Characteristics of individual biomarkers by group

Biomarker	All $n_{all} = 674$	Septic $n_s = 327$	Nonseptic $n_n = 347$	P value
Age	11.86 (27.60)	15.77 (31.95)	8.16 (22.12)	0.0004
WBC	14.04 (8.70)	15.51 (10.65)	12.66 (6.00)	<0.0001
Hgb	14.52 (3.00)	14.02 (3.15)	15.00 (2.77)	<0.0001
Hct	44.77 (12.89)	43.68 (16.04)	45.81 (8.82)	0.035
Plt	231.37 (103.38)	198.65 (106.72)	262.21 (89.80)	<0.0001
Segs	39.64 (17.25)	36.51 (17.55)	42.58 (16.42)	<0.0001
Bands	7.92 (9.61)	13.50 (11.08)	2.66 (2.60)	<0.0001
Lymph	35.47 (19.25)	30.59 (19.42)	40.07 (17.91)	<0.0001
Mono	11.09 (6.87)	11.72 (7.13)	10.50 (6.56)	0.0225
CD64	2.96 (2.42)	4.00 (2.89)	1.99 (1.22)	<0.0001

Statistical analysis of individual biomarker based on the evaluation distributions of septic group and nonseptic group. Results are presented as mean (standard deviation). P values are comparisons between septic group and nonseptic group. Any significance level of P less than 0.05 was associated with the diagnosis.
