# Supplementary material for: Which Biomarkers Reveal Neonatal Sepsis?
Source: PLoS One. 2013 Dec 18;8(12):e82700. doi: 10.1371/journal.pone.0082700 (PMC3867385; doi:10.1371/journal.pone.0082700)
Supplement: Text S1 — Supplementary Methods. (PDF) [file pone.0082700.s005.pdf]

Which Biomarkers Reveal Neonatal Sepsis? Supplementary Material

Kun Wang[1,2], Vineet Bhandari[3], Sofya Chepustanova[1], Greg Huber[4], Stephen O'Hara[1], Corey S. O'Hern[5], Mark D. Shattuck[6], Michael Kirby[1]
1 Department of Mathematics, Colorado State University, Fort Collins, CO, USA
2 Department of Mechanical Engineering & Materials Science, Yale University, New Haven, CT, USA
3 Division of Perinatal Medicine, Department of Pediatrics, Yale University School of Medicine, New Haven, CT, USA
4 Kavli Institute for Theoretical Physics, University of California, Santa Barbara, CA, USA
5 Department of Mechanical Engineering & Materials Science, Department of Applied Physics, and Department of Physics, Yale University, New Haven, CT, USA
6 Benjamin Levich Institute and Physics Department, The City College of New York, New York, NY, USA
∗ E-mail: kirby@math.colostate.edu
⋄ Referred to as (Supplementary) Text S1 in the main text.

Supplementary Methods

Univariate Analysis by Group

The canonical correlation analysis above is carried out using both sick and healthy subjects. We are able to gain additional insight into these correlations by separating the groups and exploring a univariate analysis to help us interpret the multivariate results of CCA.

Means and standard deviations (SD) of individual biomarker values were calculated for each group and all evaluations. The results, expressed as Mean (SD), are shown in Table S1. P values were also calculated in Table S1 by using two-tailed t test for the comparison between two groups. Any P value less than 0.05 was considered significant associations with sepsis diagnosis.

From Table S1, all biomarkers have univariate significance of P values such that they all have the potential diagnositic ability. Here we systematically evaluated the Pearson pairwise correlations between any pair of all biomarkers for septic group (Fig. S1(A)), nonseptic group (Fig. S1(B)).[1]

A comparison of univariate correlations between the septic group and nonseptic group (Fig. S1) illustrates some interesting points. We have observed that Bands is the sin-

[1] We comment that this approach is equivalent to CCA when each data set consists of a single variable.

gle most important biomarker for neonatal sepsis. The univariate analysis reveals that Bands and Segs actually have low correlation amongst subjects in septic group (0.08), but a notably high correlation in nonseptic group (0.41). This loss of correlation for sick subjects is potentially the reason Bands is the single most important biomarker. Note also that Bands takes on a very narrow set of values for healthy patients while it fluctuates considerably for sick individuals. See Table S1 for further details.

We also note that Hgb and Hct are highly correlated with each other in nonseptic group (0.96), but this correlation is significantly reduced for the septic group (0.46). Interestingly, we also observe that Plt and Hgb are differentially correlated with each other based on group (0.20 in nonseptic, 0.00 in septic group). CD64 also correlates very differently with Lymph based on group (0.27 in nonseptic, 0.10 in septic group).

We found that sepsis score is highly correlated both with Bands (0.56) and CD64 (0.42), which implies these two biomarkers individually are potentially good biomarkers to identify sepsis. We also found that Bands is highly correlated by Lymph (-0.46) and CD64 (0.42), which implies the Lymph and CD64 should have similar predictability with Bands. The high pairwise correlation can also be found in Hgb and Hct (0.62), WBC and Lymph(-0.51), Segs and WBC (0.44), Lymph and Segs (-0.67). These pairs of biomarkers with high correlation imply high redundant information inside pairs and may not independently associate with the sepsis diagnosis.

Based on these observations, we should not treat these biomarkers as independent diagnostic markers and a minimal set of non-redundant biomarkers should be selected to identify sepsis due to the redundant information among biomarkers.

LASSO Logistic Regression (LLR) Results

For each k, we select the k-combination set of biomarkers as identified by CCA. We construct a LLR classifier for each k from 1 to 10. We also applying LLR to all possible combinations of biomarkers for each k. We show the TPR, TNR, PPV, NPV, and ACC for the top 20 of all possible combinations in Fig. S2. It is clear that the CCA-selected biomarkers possess the best statistical measures for each k.

In Fig. S3, we show that the ROC curves become independent of k for $k \geq 5$, and thus $k = 5$ is indeed the appropriate number of biomarkers. In the inset to Fig. S3, we show the ROC curve for $k = 5$ averaged over 100 LLR models.

Mathematical Methodology

We present the mathematical theory behind CCA here connecting the statistical approach of [1] based on multivariate correlations to the geometric approach of [2] derived in terms of angles between subspaces. We connect these two ideas by showing that they give rise to the same optimization problem.

We begin with the assumption that we are trying to compare two data sets, say X and Y and that the goal is to reveal how the information in each data set is related. In our analysis the data set X is taken to be the biomarker values and the data set Y is taken to be the sepsis score. Practically speaking, the data sets X and Y are actually data matrices. The row index serves as the patient label and the column index corresponds to the measured level of a quantity of interest.[2]

We are interested in comparing how the information associated with the variables of data set X relates to the information associated with the variables comprising data set Y. To begin, we consider the statistical formulation in terms of correlation. We seek a vector a to project the rows of X onto, and a vector b to project the rows of Y onto such that the resulting values are maximally correlated over all of the rows. We adopt the convention that the rows of X and Y may be written as the column vectors x_i and y_i, respectively. Also, the colmns of X and Y will be written as the column vectors X_i and Y_j, respectively.

In other words, we write

$$X = \begin{pmatrix} | & & | & & | \\ X_1 & \ldots & X_i & \ldots & X_n \\ | & & | & & | \end{pmatrix} = \begin{pmatrix} x_1' \\ \vdots \\ x_i' \\ \vdots \\ x_m' \end{pmatrix}$$

where the prime notation denotes transpose. Equipped with this notation we seek vectors a and b with

$$\alpha_i = a' x_i$$

and

$$\beta_i = b' y_i$$

such that

$$z = \frac{\langle (\alpha - \overline{\alpha})(\beta - \overline{\beta}) \rangle}{\sqrt{\langle (\alpha - \overline{\alpha})^2 \rangle} \sqrt{\langle (\beta - \overline{\beta})^2 \rangle}}$$

For simplicity, we will assume that the data has zero mean in the sense

$$\langle x \rangle = \langle y \rangle = 0$$

from which we can then say

$$\overline{\alpha} = \overline{\beta} = 0$$

[2]We note that it is also interesting to take different sets of biomarkers as comprising X and Y but that analysis is not included here.

We would like to choose the one-dimensional subspaces spanned by a and b, respectively, such that the correlation coefficient z of α and β is maximized, in other words,

$$\max_{a,b} z$$

Using our assumption that the data has been mean subtracted we have

$$\max_{a,b} \frac{\langle a'xb'y\rangle}{\sqrt{\langle (a'x)^2\rangle\langle (b'y)^2\rangle}}$$

which can be rewritten as

$$\max_{a,b} \frac{a'\langle xy'\rangle b}{\sqrt{a'\langle xx'\rangle a}\sqrt{b'\langle yy'\rangle b}}$$

This is again equivalent to

$$\max_{a,b} \frac{a'X'Yb}{\sqrt{a'X'Xa}\sqrt{b'Y'Yb}}$$

where we have reintroduced the matrix notation.

This can be recast as the simpler optimization

$$\max_{a,b} a'X'Yb$$

subject to the contsraints

$$\|a\|_{W_X} = \|b\|_{W_Y} = 1$$

where the norms are the usual weighted Euclidean inner products with weighting matrices $X'X$ and $Y'Y$.

Before we solve this optimization problem in this form we derive an equivalent transformation which is more suitable for computation. Now we begin by writing

$$u = a_1X_1 + \cdots + a_jX_j + \cdots + a_nX_n \quad (1)$$

$$v = b_1Y_1 + \cdots + b_jY_j + \cdots + b_nY_n \quad (2)$$

So, we see that u and v live in the column spaces of the matrices X and Y, respectively.

To determine the vectors in $u \in R(X)$ and $v \in R(Y)$ that have the smallest angle between them we seek to solve

$$\max_{u\in R(X),\, v\in R(Y)} u'v$$

subject to the side condition that

$$\|u\| = \|v\| = 1$$

In other words, we seek to maximize the cosine of the angle, or equivalently, to minimize the angle between the two vectors u and v [2].

If we let

$$u = Xa, \quad v = Yb$$

then we have the equivalent problem

$$\max a'X'Yb$$

subject to

$$\|a\|_{W_X} = 1, \ \|b\|_{W_Y} = 1$$

which we recognize as being equivalent to the problem above where the correlation is being optimized in the rows.

An efficient approach to compute the solutions to this problem is to first determine the $Q - R$ decompositions for the matrices X and Y, i.e.,

$$X = Q_X R_X, \quad Y = Q_Y R_Y$$

So now we write

$$u = Q_X \phi, \quad v = Q_Y \psi$$

so the optimization problem for u and v becomes

$$\max \phi' Q_X' Q_Y \psi$$

subject to the constraints

$$\|\phi\| = \|\psi\| = 1$$

We recognize the solution to this problem as being given by the singular value decomposition

$$\Phi\Sigma\Psi = Q_X' Q_Y$$

Finally, we can compute the CCA weights using

$$a = R_X^{\dagger}\phi, \quad b = R_Y^{\dagger}\psi \qquad (3)$$

References

1. Mardia KV, Kent JT, Bibby JM (1980) Multivariate analysis .

2. Björck Å, Golub GH (1973) Numerical methods for computing angles between linear subspaces. Mathematics of computation 27: 579–594.

Supplementary Figures and Tables

This section contains the supplementary figures and tables referred to in the main text.

Table S1. Characteristics of individual biomarkers by group. Statistical analysis of individual biomarker based on the evaluation distributions of septic group and nonseptic group. Results are presented as mean (standard deviation). P values are comparisons between septic group and nonseptic group. Any significance level of P less than 0.05 was associated with the diagnosis.

Figure S1. Heatmaps of pairwise correlations magnitude. The pairwise correlations were calculated for any pair of all 10 biomarkers in septic group (A) and nonseptic group (B). The biomarkers in both x-axis and y-axis for all heatmaps are sorted ascending by the corrlation magnitude with sepsis score. The intensity of the color indicates the correlation magnitude in the pair associated with the corresponding labels of x-axis and y-axis. A high magnitude implies a strong association between two variables.

Figure S2. Exhaustive evaluation of statistical measures. The 20 highest TPR, TNR, PPV, NPV, ACC values when LLR was applied for all possible combinations of k biomarkers (blue circles) from $k = 1, \ldots, 10$. The solid red circles are the values for models built using the best k biomarkers selected by CCA.

Figure S3. Receiver operating characteristic (ROC) curves. ROC curves of TPR versus FPR for optimal sets of k biomarkers where $k = 1, \ldots, 10$ averaged over 100 LLR models. The shaded region in the inset shows the standard deviation for $k = 5$.
